# Deletions of *MGF110-9L* and *MGF360-9L* from African swine fever virus are highly attenuated in swine and confer protection against homologous challenge

**DOI:** 10.1016/j.jbc.2023.104767

**Published:** 2023-05-02

**Authors:** Dan Li, Jingjing Ren, Guoqiang Zhu, Panxue Wu, Wenping Yang, Yi Ru, Tao Feng, Huanan Liu, Jing Zhang, Jiangling Peng, Hong Tian, Xiangtao Liu, Haixue Zheng

**Affiliations:** State Key Laboratory for Animal Disease Control and Prevention, College of Veterinary Medicine, Lanzhou University, Lanzhou Veterinary Research Institute, Chinese Academy of Agricultural Sciences, Lanzhou, China

**Keywords:** African swine fever virus, MGF110-9L, MGF360-9L, Toll-like receptor 2, RNA-seq

## Abstract

African swine fever, caused by a large icosahedral DNA virus (African swine fever virus, ASFV), is a highly contagious disease in domestic and feral swine, thus posing a significant economic threat to the global swine industry. Currently, there are no effective vaccines or the available methods to control ASFV infection. Attenuated live viruses with deleted virulence factors are considered to be the most promising vaccine candidates; however, the mechanism by which these attenuated viruses confer protection is unclear. Here, we used the Chinese ASFV CN/GS/2018 as a backbone and used homologous recombination to generate a virus in which *MGF110-9L* and *MGF360-9L*, two genes antagonize host innate antiviral immune response, were deleted (ASFV-ΔMGF110/360-9L). This genetically modified virus was highly attenuated in pigs and provided effective protection of pigs against parental ASFV challenge. Importantly, we found ASFV-ΔMGF110/360-9L infection induced higher expression of Toll-like receptor 2 (TLR2) mRNA compared with parental ASFV as determined by RNA-Seq and RT-PCR analysis. Further immunoblotting results showed that parental ASFV and ASFV-ΔMGF110/360-9L infection inhibited Pam3CSK4-triggered activating phosphorylation of proinflammatory transcription factor NF-κB subunit p65 and phosphorylation of NF-κB inhibitor IκBα levels, although NF-κB activation was higher in ASFV-ΔMGF110/360-9L-infected cells compared with parental ASFV-infected cells. Additionally, we show overexpression of TLR2 inhibited ASFV replication and the expression of ASFV p72 protein, whereas knockdown of TLR2 had the opposite effect. Our findings suggest that the attenuated virulence of ASFV-ΔMGF110/360-9L might be mediated by increased NF-κB and TLR2 signaling.

African swine fever virus (ASFV) is the causative agent of African swine fever (ASF), a contagious and often fatal disease of domestic pigs and wild boar. ASFV has a complicated architecture with a linear, double-stranded DNA genome (∼170–193 kb) containing 150 to 167 ORFs (1, 2, 3). Currently, ASF is endemic in more than 20 sub-Saharan African countries with serious economic consequences for the swine industry (3, 4). The disease spread from Africa to the Caucasus, especially within the country of Georgia in 2007 (5). Afterward, ASF spread to most countries in the eastern part of the European Union in 2014. In August 2018, the ASF epidemic started in China and it has caused huge economic losses to the pig industry (6).

Different vaccine strategies for ASF have been evaluated in the past decades. However, due to the huge ASFV genome and complex mutation mechanism, no clinically effective vaccines have been developed to date (7), including inactivated vaccines, DNA vaccines, subunit vaccines, and adenovirus-vectored vaccines (8, 9, 10, 11, 12). Rational development of attenuated strains by genetic manipulation is a valid alternative, and perhaps a safer methodology, compared to the use of naturally attenuated isolates (3, 13). Some attenuated ASFV strains, obtained by genetic manipulation, consisting of deletions of single genes or a group of genes, have shown to induce protection against the virulent parental virus (3, 14, 15, 16, 17, 18, 19, 20, 21, 22); however, the mechanism of attenuation and protection is unclear.

Our previous research showed that the survival rate of pigs inoculated with low doses of ASFV MGF110-9L–deleted viruses (23) and ASFV MGF360-9L–deleted viruses (24) was 60% and 80%, respectively. Here, we report the construction of a recombinant virus (ASFV-ΔMGF110/360-9L) derived from the highly virulent ASFV CN/GS/2018 isolate by deleting the *MGF110-9L* and *MGF360-9L* genes. Pigs inoculated with a high dose of ASFV-ΔMGF110/360-9L remained clinically normal and were effectively protected when challenged with the virulent parental ASFV. Besides, Toll-like receptor 2 (TLR2) mRNA levels increased in ASFV-ΔMGF110/360-9L–infected primary porcine alveolar macrophages (PAMs) compared with in parental ASFV-infected PAMs. Notably, both of parental ASFV and ASFV-ΔMGF110/360-9L infection inhibited TLR2 signaling, whereas ASFV-ΔMGF110/360-9L weakened this inhibitory effect compared with parental ASFV. These findings suggest that the attenuated virulence of ASFV-ΔMGF110/360-9L might be mediated by weakening the inhibitory effect of ASFV-ΔMGF110/360-9L on TLR2 signaling.

## Results

### Construction of ASFV-ΔMGF110/360-9L virus

**Figure 1:**
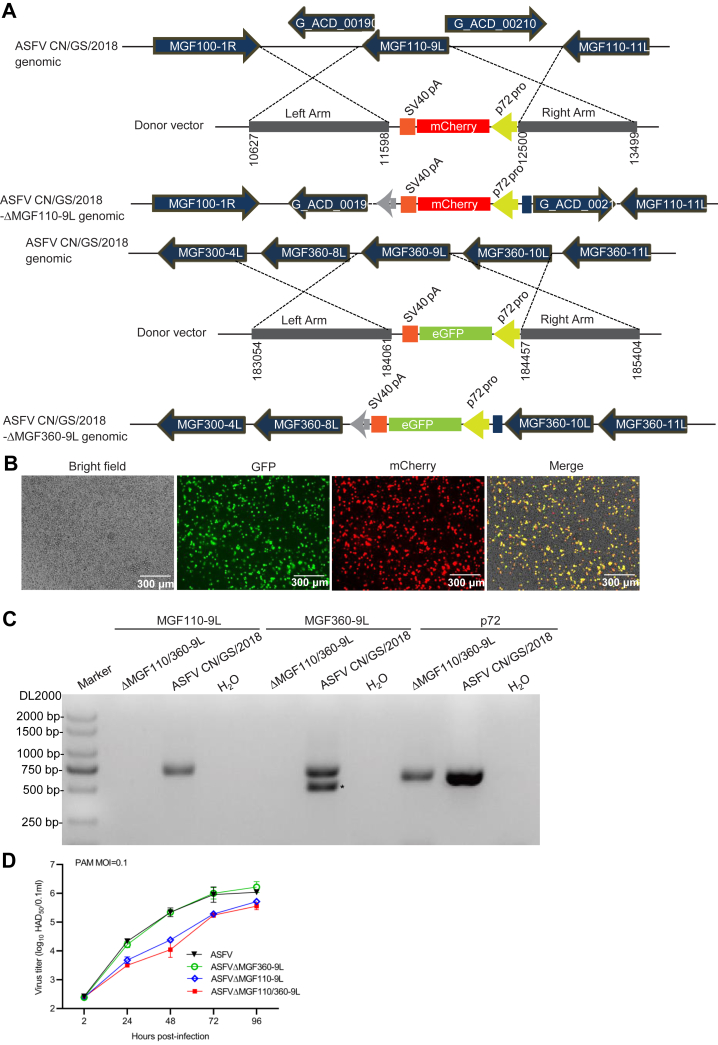
**Construction of ASFV-ΔMGF110/360-9L.***A*, schematic representation of the gene(s) and region(s) deleted in each gene-deleted ASFV. The deleted gene segments were replaced with the p72 eGFP or p72 mCherry reporter gene cassette as indicated. Nucleotide positions indicating the boundaries of the deletion relative to the ASFV CN/GS/2018 genome are indicated. *B*, fluorescence of virus-infected primary porcine alveolar macrophages. Scale bar represents 300 μm. *C*, PCR analysis of ASFV-ΔMGF110/360-9L using specific primers targeting *MGF110-9L*, *MGF360-9L*, or p72 (*B646L*) genes. ∗, nonspecific band. *D*, multistep virus growth curves of ASFV-ΔMGF110-9L, ASFV-ΔMGF360-9L, ASFV-ΔMGF110/360-9L, and parental ASFV CN/GS/2018. Primary swine macrophage cell cultures were infected (MOI = 0.1) with each of the viruses and virus titers at the indicated times postinfection. The graphs show the mean ± SD. In the displayed experiment, three replicates were included per experimental condition. MOI, multiplicity of infection; ASFV, African swine fever virus.

**Supporting Figure S1:**
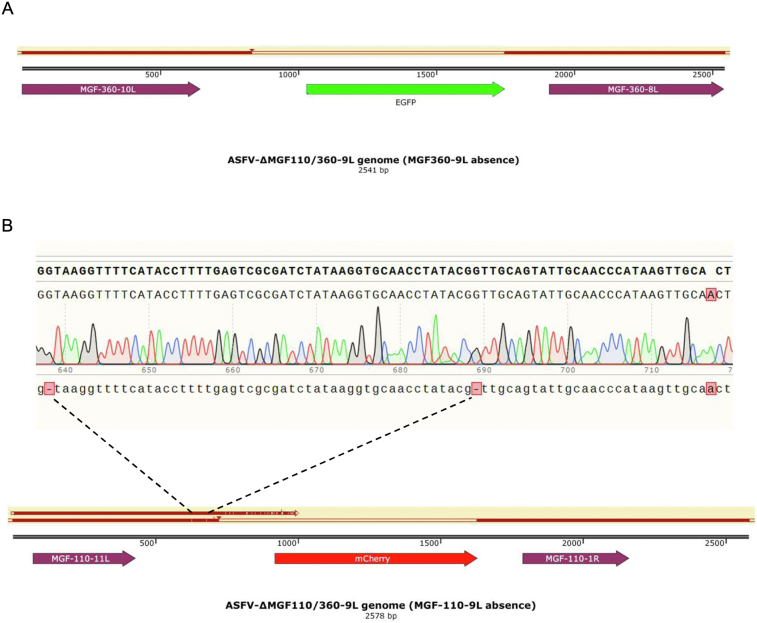
**Sequenced results of ASFV-ΔMGF110/360-9L genome covering the modified regions.** ASFV-ΔMGF110/360-9L genome was isolated from virus stock and the modified region was amplified by PCR utilizing primers MGF-360-9L-F/R and MGF-110-9L-F/R. Afterwards, the PCR results were sequenced to confirm the absence of MGF-360-9L gene (*A*) and MGF-110-9L gene (*B*).Note: In Fig. S1*B*, two gaps and one redundant base were found in ASFV-ΔMGF110/360-9L genome. However, the peak map of sequencing result revealed that these results were caused by the sequencing quality. Therefore, these results were corrected by analyzing the peak map of sequencing. After correction, no gaps or mismatches were observed in ASFV-ΔMGF110/360-9L genome.

ASFV-encoding genes *MGF110-9L* (23) and *MGF360-9L* (25) have been identified as the virulence factor of ASFV. To further determine the combined roles of *MGF110-9L* with *MGF360-9L* during ASFV infection in cell cultures and the virulence and pathogenicity in swine, a recombinant virus (ASFV-ΔMGF110/360-9L) lacking the *MGF110-9L* and *MGF360-9L* genes was constructed from a highly pathogenic genotype II virus (ASFV CN/GS/2018) using homologous recombination. The *MGF110-9L* and *MGF360-9L* genes were replaced by a cassette containing the fluorescent gene mCherry or eGFP, respectively, under the ASFV p72 promoter (Fig. 1*A*). The recombinant virus was obtained after ten successive rounds of limiting dilution purification based on fluorescence activity. The recombinant virus obtained from the last round of purification was amplified in PAMs to obtain a virus stock. Red and green fluorescence were observed in ASFV-ΔMGF110/360-9L–infected PAM cells at 12 h postinfection (Fig. 1*B*). The absence of *MGF110-9L* and *MGF360-9L* genes in the ASFV-ΔMGF110/360-9L mutant virus was confirmed by sequence analysis of both the parental and mutant viruses and confirmed by PCR. *MGF110-9L* or *MGF360-9L* genes were detected in ASFV-infected PAMs but not in ASFV-ΔMGF110/360-9L–infected PAMs, and the p72 gene was detected in ASFV-infected PAMs and ASFV-ΔMGF110/360-9L–infected PAMs (Figs. 1*C* and S1). Collectively, these results indicate that the ASFV-ΔMGF110/360-9L mutant virus was successfully constructed.

*In vitro* growth characteristics of ASFV-ΔMGF110/360-9L were assessed in PAMs and compared relative to parental ASFV, ASFV-ΔMGF110-9L, as well as ASFV-ΔMGF360-9L in multistep growth curves. PAMs were infected with the indicated virus at an multiplicity of infection (MOI) of 0.1, and samples were collected at 2, 24, 48, 72, and 96 h postinfection. ASFV-ΔMGF110/360-9L or ASFV-ΔMGF110-9L displayed a growth kinetic dramatically delayed when compared to parental ASFV or ASFV-ΔMGF360-9L (Fig. 1*D*).

### Assessment of ASFV-ΔMGF110/360-9L virulence in swine

**Figure 2:**
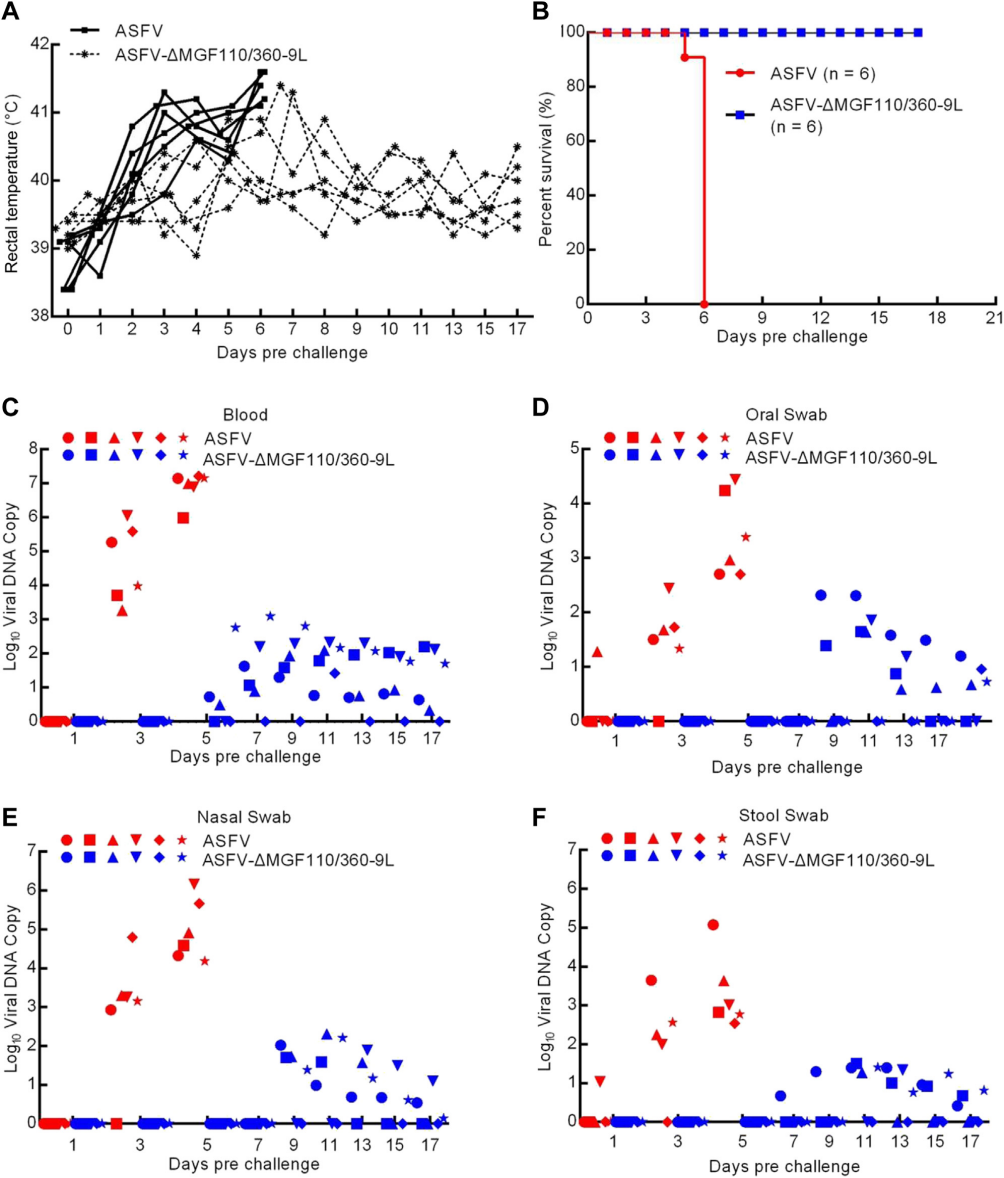
**Virulence evaluation of ASFV-ΔMGF110/360-9L in pigs.***A*, body temperature of pigs inoculated with either 10^4^ median hemadsorption units (HAD_50_) of ASFV CN/GS/2018 (*solid lines*) or ASFV-ΔMGF110/360-9L (*dashed lines*). *B*, survival rates of pigs inoculated with parental ASFV CN/GS/2018 or ASFV-ΔMGF110/360-9L. *C*–*F*, viral DNA copies in (*C*) blood, (*D*) oral swab, (*E*) nasal swab, or (*F*) stool swab of pigs. Each *shape* represents an individual animal from each group. ASFV, African swine fever virus.

To determine the effect of *MGF110-9L*/*MGF360-9L* deletion in swine, 30 to 40 kg pigs were inoculated *via* intramuscular injection (i.m.) with 10^4^ median hemadsorption units (HAD_50_) of either ASFV-ΔMGF110/360-9L (n = 6) or ASFV CN/GS/2018 (n = 6). As expected, pigs infected with 10^4^ HAD_50_ of parental ASFV exhibited increased body temperature (>40 °C) by day 3 to 4 postinfection (dpi), presenting with clinical signs related with the disease including anorexia, depression, purple skin discoloration, staggering gait, and diarrhea. Signs of the disease aggravated progressively over time and pigs died within 7 dpi (Fig. 2, *A* and *B*). By contrast, six pigs inoculated with ASFV-ΔMGF110/360-9L, which survived the 17-days observation period, developed fever for only a short time and then their temperature dropped below 40.5 °C (Fig. 2, *A* and *B*). Therefore, compared to its parental ASFV, the virulence of ASFV-ΔMGF110/360-9L is significantly reduced.

Viremia was quantified several days postinfection in experimentally inoculated pigs using quantitative PCR (qPCR) analysis. As expected, pigs inoculated with 10^4^ HAD_50_ of virulent parental ASFV exhibited higher blood viral titers (10^3.2^–10^7.2^ copies) until the day of their death compared with that of pigs injected with ASFV-ΔMGF110/360-9L (10^0.5^–10^3^ copies) (Fig. 2*C*). Similarly, high titers were observed in pigs inoculated with 10^4^ HAD_50_ parental ASFV in oral (Figs. 2*D*, 10^1.3^–10^4.4^ copies), nasal (Fig. 2*E*, 10^2.9^–10^6.2^ copies), and stool swab (Fig. 2*F*, 10–10^5^ copies). Conversely, pigs injected with mutant ASFV-ΔMGF110/360-9L had relatively low viral DNA copy numbers in oral (Fig. 2*D*, 10^0.6^–10^2.3^ copies), nasal (Fig. 2*E*, 10^0.2^–10^2.2^ copies), and stool swab (Fig. 2*F*, 10^0.4^–10^1.5^ copies) samples compared with those of parental ASFV-inoculated pigs. These results suggested that the deletions of *MGF110-9L* and *MGF3*6*0-9L* weaken the virulence of the parental ASFV strain in pigs.

### Protective efficacy of ASFV-ΔMGF110/360-9L against a challenge with lethal parental ASFV strain

**Figure 3:**
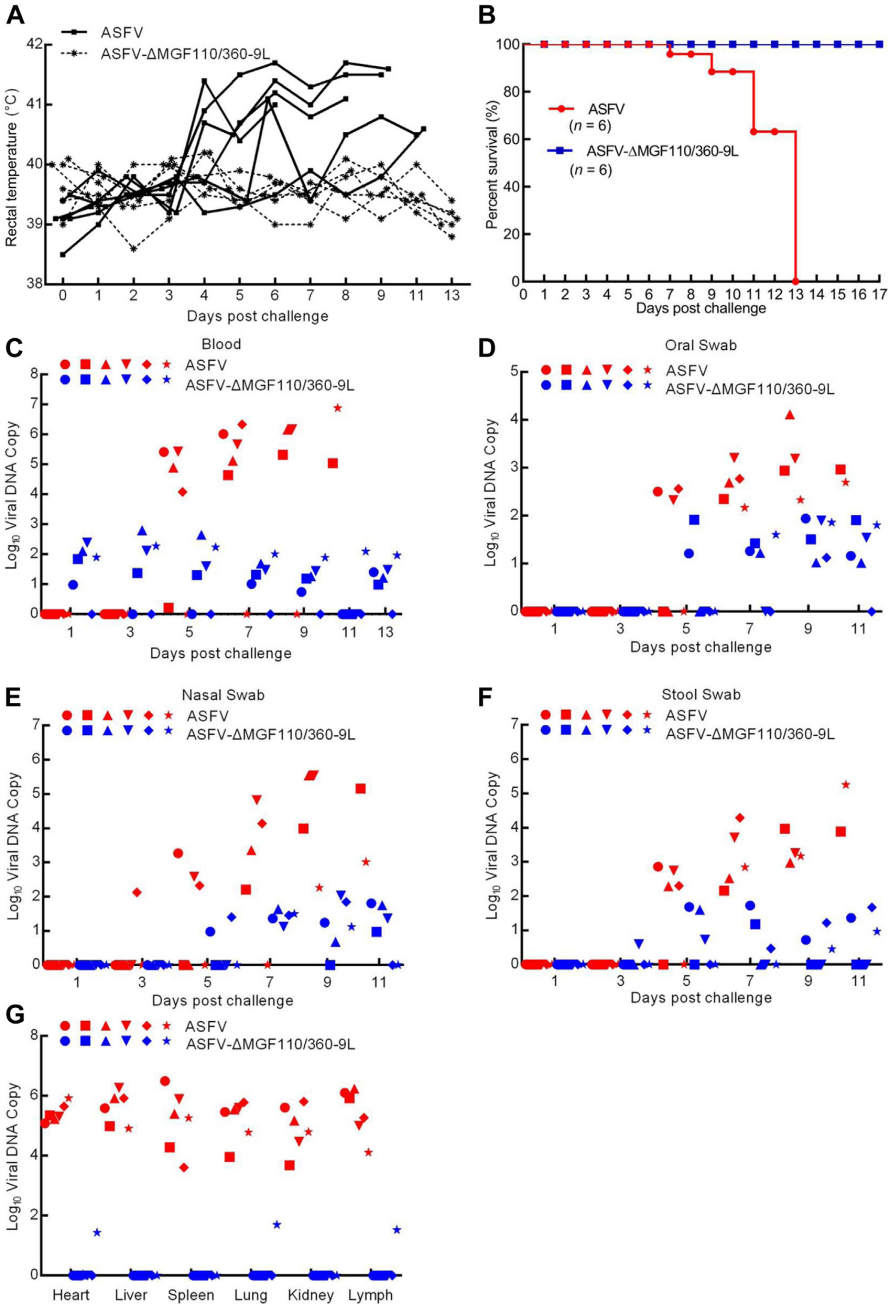
**Protective efficacy of pigs induced by ASFV-ΔMGF110/360-9L.** Groups of pigs inoculated with 10^4^ median hemadsorption units (HAD_50_) of the indicated gene-deleted ASFV were challenged intramuscularly with parental ASFV CN/GS/2018. Samples were collected from dead pigs or surviving pigs that were euthanized on day 13 postchallenge for virus DNA detection. Days postchallenge refers to untreated pigs inoculated with either 10^4^ HAD_50_ of ASFV CN/GS/2018 or the time after a secondary challenge with 10^2^ HAD_50_ ASFV CN/GS/2018 from pigs initially challenged with ASFV-ΔMGF110/360-9L for 17 days (shown in Fig. 2). *A*, body temperature of pigs. *B*, survival rates of pigs. *C*–*G*, viral DNA copies in (*C*) blood, (*D*) oral swab, (*E*) nasal swab, (*F*) stool swab, or (*G*) tissues of the pigs, with each *shape* representing an individual animal from each group. ASFV, African swine fever virus.

To assess the protective effect of the ASFV-ΔMGF110/360-9L–infected pigs against a challenge with the lethal parental ASFV strain, the group of pigs inoculated with 10^4^ HAD_50_ of ASFV-ΔMGF110/360-9L and six previously untreated pigs (control) were i.m. challenged days later with 10^2^ HAD_50_ of the parental virus. The control pigs displayed ASF-related signs at 4 days postchallenge (p.c.), with clinical signs evolving into more severe manifestations in the following days, and all control pigs euthanized within 13 days p.c. (Fig. 3, *A* and *B*). Conversely, pigs in the group infected with ASFV-ΔMGF110/360-9L survived and remained healthy during the duration of the observation period after challenge with the virulent parental virus (Fig. 3, *A* and *B*).

**Figure 4:**
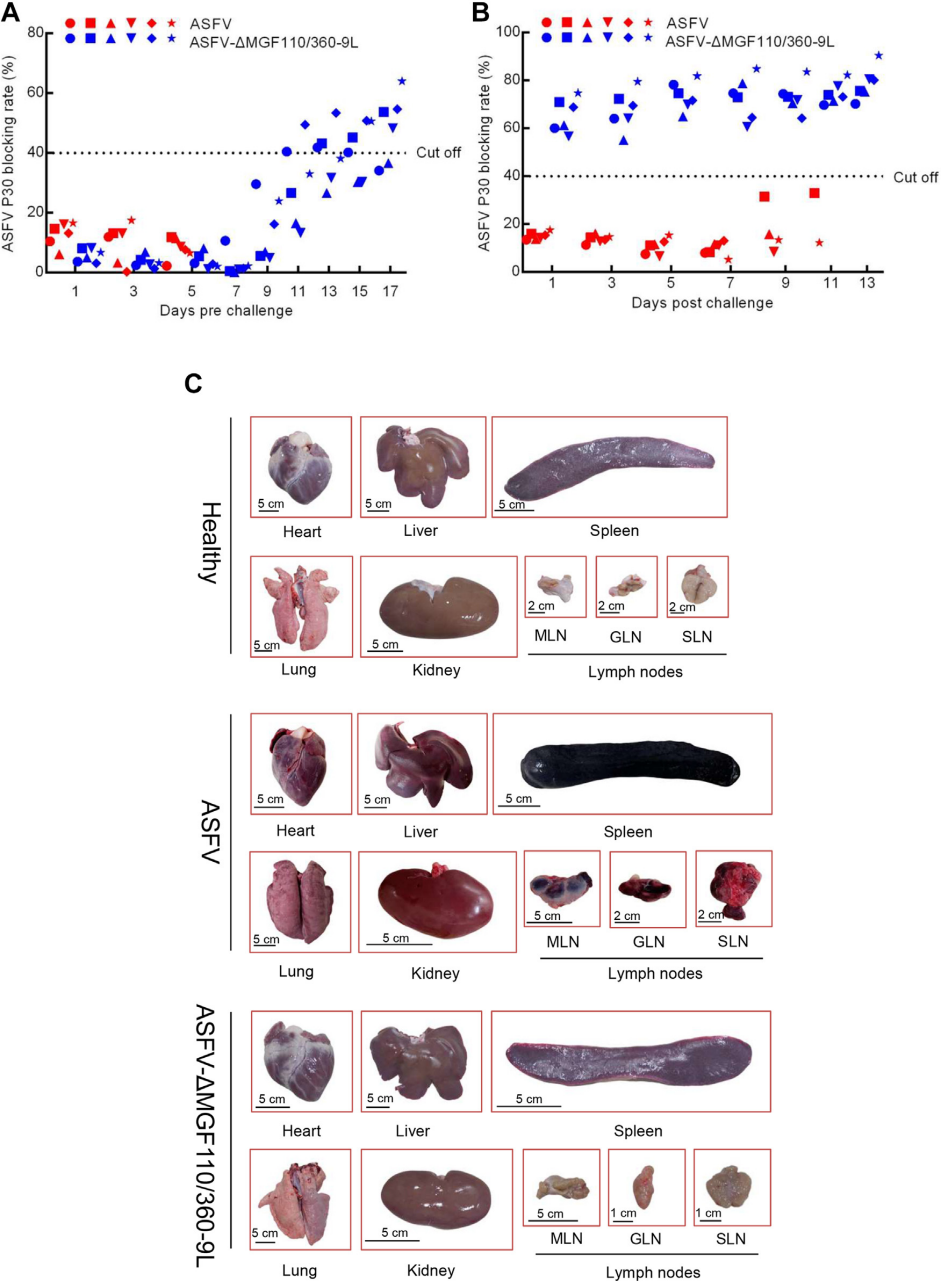
**Detection of ASFV p30 by blocking ELISA.***A*, anti-p30 antibody detected by blocking ELISA in pigs intramuscularly inoculated with 10^4^ median hemadsorption units (HAD_50_) of ASFV-ΔMGF110/360-9L or ASFV CN/GS/2018 for 17 days,\ and (*B*) after secondary challenge with 10^2^ HAD_50_ of ASFV CN/GS/2018. Each *shape* represents an individual animal from each group. *C*, gross pathological changes of heart, liver, spleen, lung, kidney, and lymph nodes in healthy, ASFV challenged and ASFV-ΔMGF110/360-9L vaccinated pigs. MLN, mesenteric lymph node; GLN, gastrohepatic lymph node; SLN, submandibular lymph node. ASFV, African swine fever virus.

As expected, viremia titers in the control pigs challenged with parental ASFV were high (ranging between 10^4.6^ and 10^6.3^ copies) on day 7, remaining high (10^5^ copies) until the pigs were euthanized (Fig. 3*C*). By contrast, the viremia values (10^0.7^–10^2.8^ copies) of the ASFV-ΔMGF110/360-9L–infected pigs progressively decreased during the observed period (Fig. 3*C*). Similarly, viral DNA copies in the oral (10^2.3^–10^4^ copies in control group, 10^1.1^–10^2^ copies in ASFV-ΔMGF110/360-9L group), nasal (10^2.1^–10^5.5^ copies in control group, 10^0.6^–10^2^ copies in ASFV-ΔMGF110/360-9L group), and stool swabs (10^2.3^–10^5.2^ copies in control group, 10^0.4^–10^1.7^ copies in ASFV-ΔMGF110/360-9L group) of ASFV-ΔMGF110/360-9L–infected pigs after challenge with the virulent ASFV were lower than those of challenged control pigs (Fig. 3, *D*–*F*). Furthermore, viral DNA copy numbers in the heart (10^5^–10^5.9^ copies in control group, 0–10^1.4^ copies in ASFV-ΔMGF110/360-9L group), liver (10^4.9^–10^6.3^ copies in control group, undetectable in ASFV-ΔMGF110/360-9L group), spleen (10^3.6^–10^6.5^ copies in control group, undetectable in ASFV-ΔMGF110/360-9L group), lung (10^4^–10^5.8^ copies in control group, 0–10^1.7^ copies in ASFV-ΔMGF110/360-9L group), kidney (10^.3.7^–10^5.8^ copies in control group, undetectable in ASFV-ΔMGF110/360-9L group), and lymph (10^4^–10^6.2^ copies in control group, 0–10^1.5^ copies in ASFV-ΔMGF110/360-9L group) of ASFV-ΔMGF110/360-9L–infected pigs were significantly lower than those in parental ASFV-infected pigs (Fig. 3*G*). Notably, the pathological changes showed that lymph nodes and spleen from ASFV-infected group were consistently hemorrhagic and enlarged, whereas it showed white/pink in color without inflammation in the ASFV-ΔMGF110/360-9L–vaccinated pigs (Fig. 4*C*). As ASFV replication also occurred at low levels in heart, liver, lung, and kidney samples from ASFV-ΔMGF110/360-9L–vaccinated pigs, the degrees of these tissue involvements and resulting tissue damage were much less severe (Fig. 4*C*). Collectively, these data indicate that inoculation with ASFV-ΔMGF110/360-9L protected pigs against ASFV CN/GS/2018 lethal challenge.

### Host antibody response in pigs infected with ASFV-ΔMGF110/360-9L

Previous study indicated that the level of circulating antibodies is the only immunological parameter constantly associated with protection against challenge (3). We measured the specific antibody response induced by ASFV-ΔMGF110/360-9L. The titers of anti-ASFV p30–circulating antibodies gradually increased from days 7 to 17 in ASFV-ΔMGF110/360-9L–infected pigs, whereas the levels of p30 antibody did not present any change in ASFV-infected pigs until death (Fig. 4*A*). ASFV-ΔMGF110/360-9L–infected pigs challenged with the highly virulent parental ASFV isolate presented the higher levels of ASFV p30 antibody, whereas control pigs showed the lower levels of ASFV p30 antibody (Fig. 4*B*). These results indicated ASFV-ΔMGF110/360-9L induced the production of the anti-ASFV antibodies, and the anti-ASFV antibodies might confer protection against the challenge.

### Transcriptome analysis of ASFV-ΔMGF110/360-9L– and ASFV-infected PAMs

**Figure 5:**
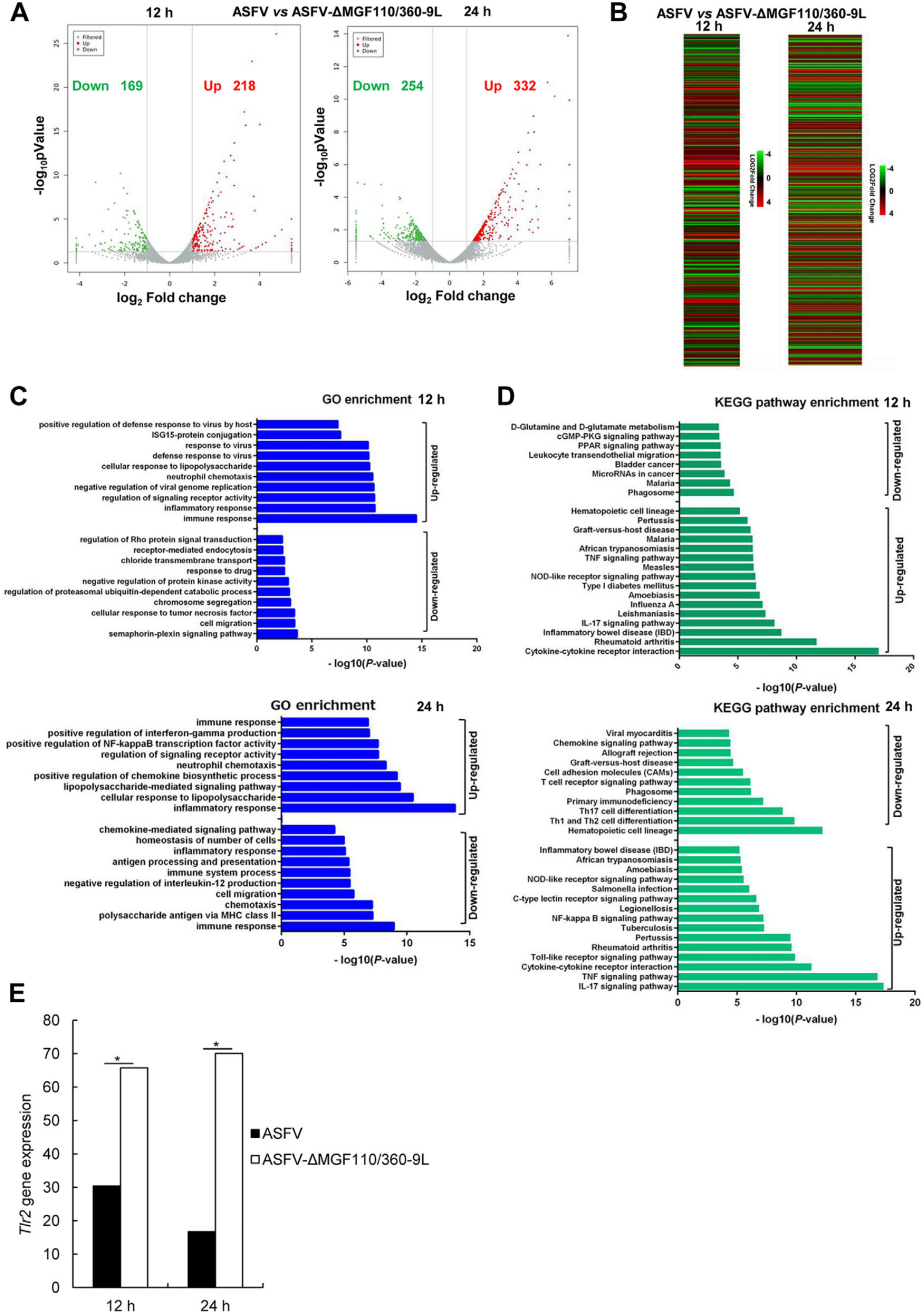
**Transcriptome profiles of primary porcine alveolar macrophages infected with ASFV CN/GS/2018 or ASFV-ΔMGF110/360-9L (MOI = 0.1) at 12 or 24 h.***A*, volcano map of differentially expressed genes (DEGs) in PAMs infected with ASFV CN/GS/2018 or ASFV-ΔMGF110/360-9L. *Red dots* indicate significantly upregulated DEGs, *green dots* indicate significantly downregulated DEGs, and *gray dots* indicate no significant change. *B*, heat map of DEGs in PAMs infected with ASFV CN/GS/2018 or ASFV-ΔMGF110/360-9L. *C*, GO terms at 12 h (*top* panel) and 24 h (*bottom* panel). *D*, KEGG pathway enrichment at 12 h (*top* panel) and 24 h (*bottom* panel). *E*, *Tlr2* gene expression of PAMs infected with ASFV CN/GS/2018 (*filled bar*) or ASFV-ΔMGF110/360-9L (*unfilled bar*). The graphs show the mean ± SD. In the displayed experiment, three replicates were included per experimental condition. ∗*p* < 0.05, represent the ASFV and ASFV-ΔMGF110/360-9L groups. ASFV, African swine fever virus; GO, gene ontology; KEGG, Kyoto Encyclopedia of Genes and Genomes; PAM, porcine alveolar macrophage; MOI, multiplicity of infection; TLR2, Toll-like receptor 2.

The volcano and heat maps show that there were 169 downregulated differentially expressed genes (DEGs) and 218 upregulated DEGs between ASFV- and ASFV-ΔMGF110/360-9L–infected PAMs at 12 h (Fig. 5, *A* and *B*). Meanwhile, 254 downregulated DEGs and 332 upregulated DEGs were screened between ASFV- and ASFV-ΔMGF110/360-9L–infected PAM cells at 24 h (Fig. 5, *A* and *B*). Gene ontology (GO) analysis showed that these upregulated DEGs were mainly enriched in immune response, inflammatory response, and cellular response to lipopolysaccharide (Fig. 5*C*). Downregulated DEGs were mainly enriched in immune response, cell migration, and polysaccharide antigen *via* MHC class II (Fig. 5*C*). In addition, Kyoto Encyclopedia of Genes and Genomes pathway analysis showed that DEGs were enriched in interleukin (IL)-17 signaling pathway, T cell receptor signaling pathway, tumor necrosis factor (TNF) signaling pathway, and cytokine–cytokine receptor interaction (Fig. 5*D*). Intriguingly, we observed significant *Tlr2* gene upregulation in ASFV-ΔMGF110/360-9L–infected PAMs compared with that in ASFV-infected PAMs at 12 and 24 h based on RNA-Seq analysis (Fig. 5*E*).

### ASFV-ΔMGF110/360-9L–infected PAMs enhanced TLR2 signaling compared with ASFV-infected PAMs

**Figure 6:**
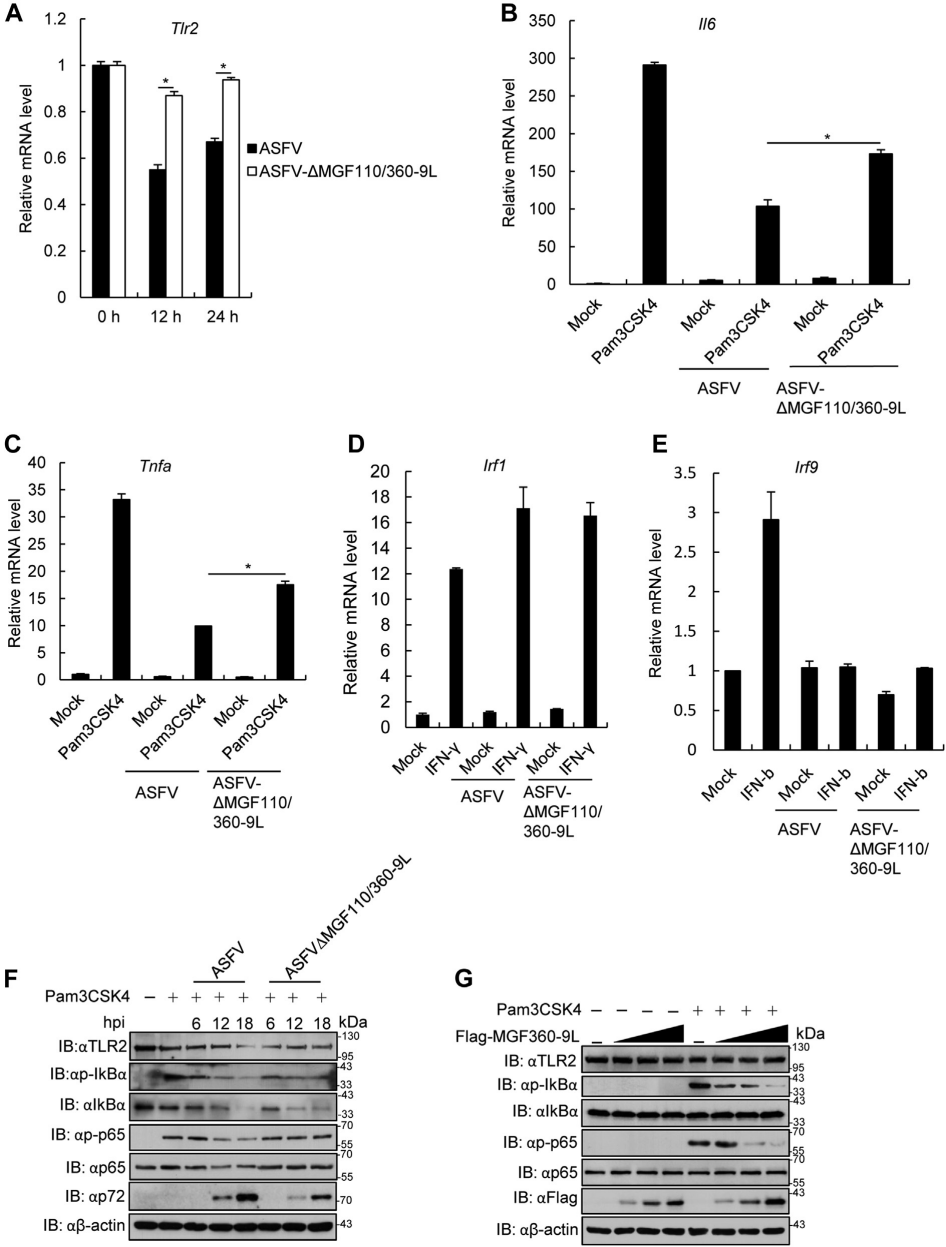
**ASFV-ΔMGF110/360-9L increased Toll-like receptor 2 mRNA level and Pam3CSK4-triggered TLR2 signaling.***A*, ASFV-ΔMGF110/360-9L-triggered transcription of *Tlr2* in primary porcine alveolar macrophages (PAMs). PAMs were infected with ASFV CN/GS/2018 or ASFV-ΔMGF110/360-9L (MOI = 0.1) for the indicated times followed by RT-PCR analysis. The graphs show the mean ± SD. In the displayed experiment, three replicates were included per experimental condition. ∗*p* < 0.05, represent the ASFV and ASFV-ΔMGF110/360-9L groups. *B* and *C*, PAMs were infected with ASFV CN/GS/2018 or ASFV-ΔMGF110/360-9L (MOI = 0.1) for 12 h or uninfected (mock), and then the cells were treated or not with Pam3CSK4 (100 ng/ml) for 4 h followed by RT-PCR analysis of (*B*) *Il6* or (*C*) *Tnfa* expression. The graphs show the mean ± SD. In the displayed experiment, three replicates were included per experimental condition. ∗*p* < 0.05, represent the ASFV- and ASFV-ΔMGF110/360-9L-Pam3CSK4–treated groups. *D*, effect of ASFV CN/GS/2018 or ASFV-ΔMGF110/360-9L infection on IFN-γ–triggered *Irf1* transcription in PAMs. PAMs were infected or not with ASFV CN/GS/2018 or ASFV-ΔMGF110/360-9L for 12 h and then treated or not with IFN-γ (100 ng/ml) for 4 h followed by RT-PCR analysis. *E*, effect of ASFV CN/GS/2018 or ASFV-ΔMGF110/360-9L infection on IFN-β–triggered *Irf9* transcription in PAMs. PAMs were infected or not with ASFV CN/GS/2018 or ASFV-ΔMGF110/360-9L for 12 h and then treated or not with IFN-β (100 ng/ml) for 4 h followed by RT-PCR analysis. *F*, effect of ASFV CN/GS/2018 or ASFV-ΔMGF110/360-9L on Pam3CSK4-triggered phosphorylation of IκBα or p65 in PAMs. The experiments were performed as described for (*B*). *G*, effect of MGF360-9L gene on TLR2 signaling *in vitro*. The iPAM cells were transfected with Flag-MGF-360-9L plasmid (0, 1, 2, and 4 μg) for 20 h and then treated with Pam3CSK4 (100 ng/ml) for another 4 h. The cell lysates were analyzed by immunoblotting utilizing the indicated antibodies. ASFV, African swine fever virus; IFN, interferon; RT-PCR, real time PCR; TLR2, Toll-like receptor 2; MOI, multiplicity of infection; TNF, tumor necrosis factor.

**Supporting Figure S2:**
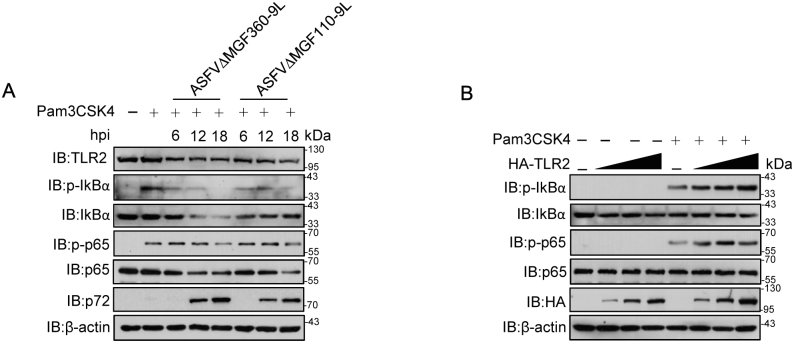
*A*, effect of ASFV-ΔMGF360-9L or ASFV-ΔMGF110-9L on Pam3CSK4-triggered phosphorylation of IκBα or p65 in primary porcine alveolar macrophages (PAMs). PAMs were infected with ASFV-ΔMGF360-9L or on Pam3CSK4-triggered phosphorylation of (MOI = 0.1) for 6, 12, and 18 h or uninfected (mock), and then the cells were treated or not with Pam3CSK4 (100 ng/ml) for another 4 h. The cell lysates were analyzed by immunoblotting utilizing the indicated antibodies. *B*, effect of TLR2 on TLR2 signaling *in vitro*. The iPAM cells were transfected with HA-TLR2 plasmid (0, 1, 2 and 4 μg) for 20 h, and then treated with Pam3CSK4 (100 ng/ml) for another 4 h. The cell lysates were analyzed by immunoblotting utilizing the indicated antibodies.

RT-PCR results confirmed that *Tlr2* mRNA expression was significantly upregulated in ASFV-ΔMGF110/360-9L–infected PAMs compared with that in ASFV-infected PAMs (Fig. 6*A*). The effect of ASFV-ΔMGF110/360-9L on TLR2 signaling was evaluated by measuring the mRNA expression of IL6 and TNFα in ASFV-ΔMGF110/360-9L– or ASFV-infected PAMs treated with Pam3CSK4. Both ASFV-ΔMGF110/360-9L– and ASFV-infected PAMs inhibited Pam3CSK4-triggered expression of *IL6* and *TNFα* mRNA, but the levels of *IL6* and *TNFα* were significantly higher in ASFV-ΔMGF110/360-9L–infected and Pam3CSK4-treated PAMs than that in ASFV-infected PAMs (Fig. 6, *B* and *C*). Meanwhile, ASFV-ΔMGF110/360-9L– or ASFV-infected PAMs treated with interferon (IFN)-γ or IFN-β resulted in unchanged levels of the IFN regulatory factor (IRF) 1 or IRF9 genes (Fig. 6, *D* and *E*). In addition, Pam3CSK4-triggered p-p65 and phosphorylated nuclear factor kappa (p-IκBα) levels in ASFV-ΔMGF110/360-9L–infected PAMs were higher than these in ASFV-infected PAMs (Fig. 6*F*). Furthermore, we observed that both of ASFV-ΔMGF110-9L and ASFV-ΔMGF360-9L infection reduced Pam3CSK4-triggered p-p65 and p-IκBα levels (Fig. S2A). Notably, MGF360-9L overexpression inhibited Pam3CSK4-triggered p-p65 and p-IκBα levels at a dose-dependent manner in iPAM cells (Fig. 6*G*). By contrast, TLR2 overexpression enhanced Pam3CSK4-triggered p-p65 and p-IκBα levels in iPAM cells (Fig. S2*B*). Taken together, ASFV-ΔMGF110/360-9L enhanced Pam3CSK4-induced TLR2 signaling compared with parental ASFV.

### TLR2 inhibited ASFV replication in PAMs

**Figure 7:**
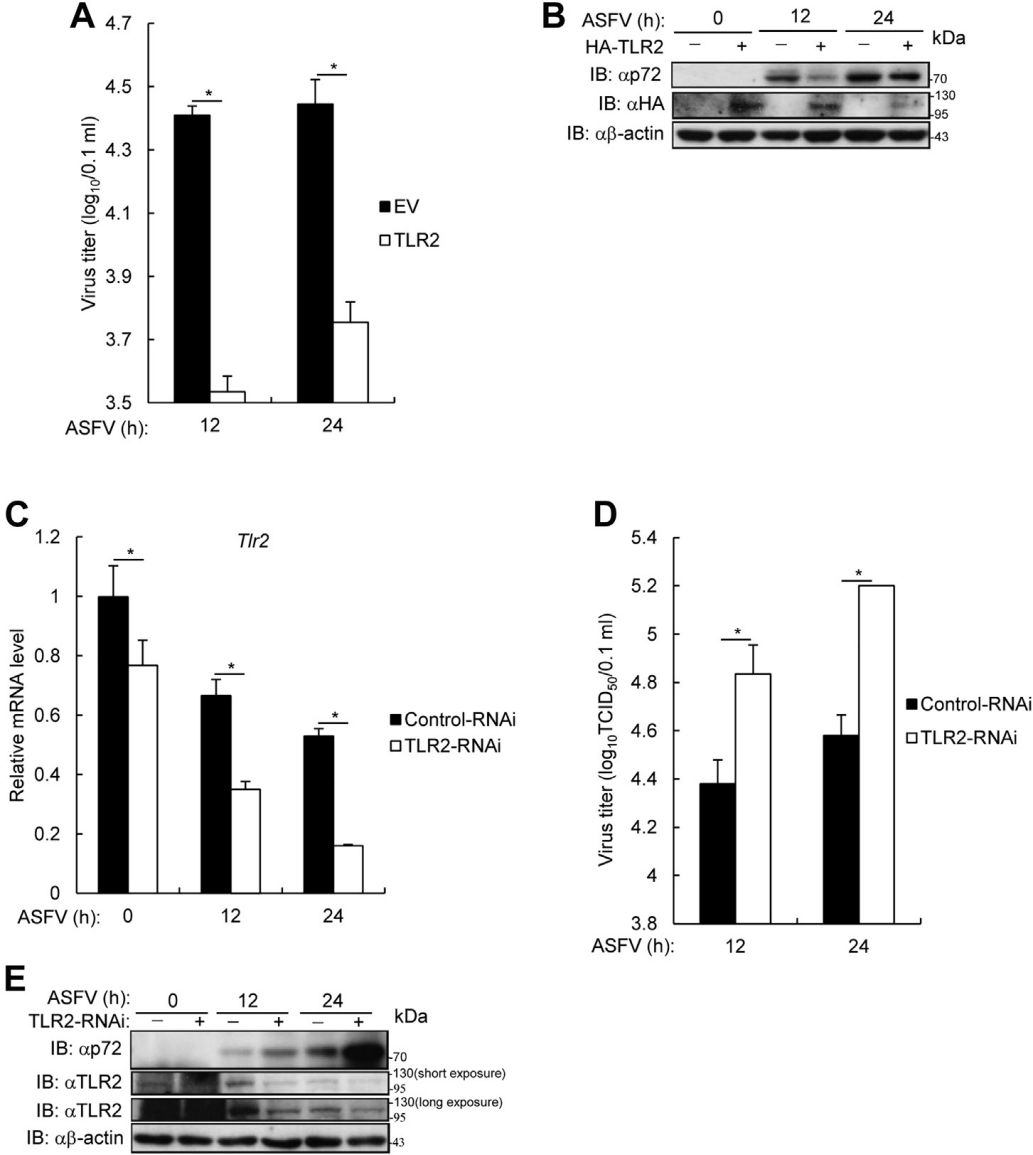
**Effect of Toll-like receptor 2 on ASFV titer and ASFV p72 protein.***A* and *B*, MA-104 cells were transfected with TLR2 plasmid for 24 h, followed by infection with ASFV for the indicated time. *A*, ASFV titer and (*B*) ASFV p72 expression level in TLR2-overexpressed MA-104 cells. The graphs show the mean ± SD. In the displayed experiment, three replicates were included per experimental condition. ∗*p* < 0.05, represent the EV- and TLR2-overexpressed groups. *C*–*E*, primary porcine alveolar macrophages (PAMs) were transfected with control-RNAi or TLR2-RNAi by Lipofectamine 2000 for 48 h. PAMs were infected with ASFV (MOI = 0.1) for the indicated times followed by RT-PCR analysis. *C*, *TLR2* levels in TLR2-knockdown PAMs infected with ASFV. The graphs show the mean ± SD. In the displayed experiment, three replicates were included per experimental condition. ∗*p* < 0.05, represent the control-RNAi– and TLR2-RNAi–silenced groups. *D*, ASFV titer of TLR2-knockdown PAM cells. The graphs show the mean ± SD. In the displayed experiment, three replicates were included per experimental condition. ∗*p* < 0.05, represent the control-RNAi– and TLR2-RNAi–silenced groups. *E*, ASFV p72 protein in TLR2-knockdown PAMs. ASFV, African swine fever virus; RT-PCR, real time PCR; RNAi, interfering RNA; MOI, multiplicity of infection; TLR2, Toll-like receptor 2.

**Supporting Figure S3:**
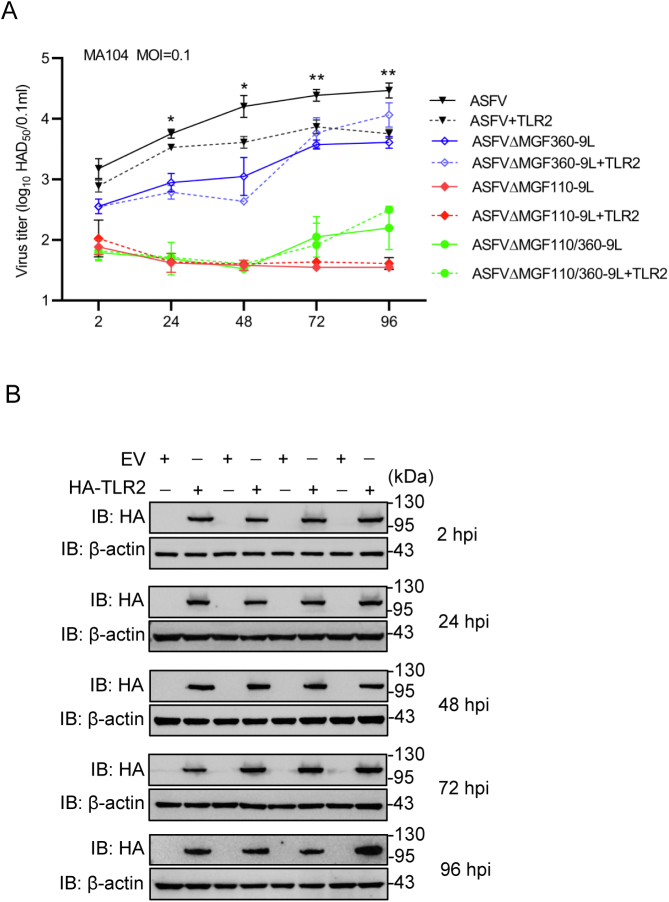
**Effect of TLR2 overexpression on the replication of ASFV, ASFVΔMGF360-9L, ASFVΔMGF110-9L and ASFVΔMGF110/360-9L.***A*, MA-104 cells were transfected with TLR2 plasmid for 24 h, and then infected with ASFV, ASFVΔMGF360-9L, ASFVΔMGF110-9L and ASFVΔMGF110/360-9L at a MOI of 0.1 for the indicated time points. Virus titers were measured by HAD_50_ assay. *B*, TLR2 overexpression at the indicated time points was analyzed by immunoblotting assay.

**Supporting Figure S4:**
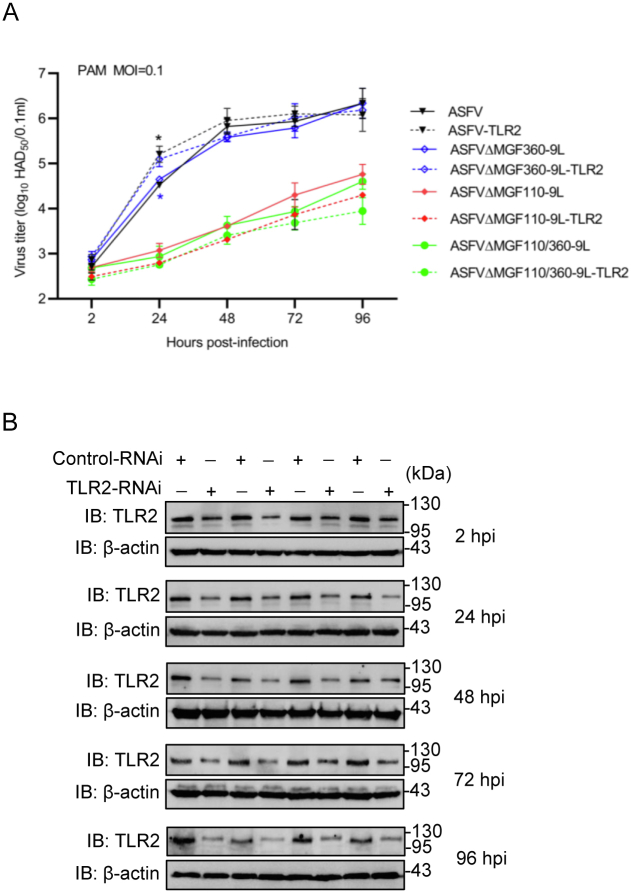
**Effect of TLR2 knockdown on the replication of ASFV, ASFVΔMGF360-9L, ASFVΔMGF110-9L and ASFVΔMGF110/360-9L.***A*, PAM cells were silenced with TLR2-RNAi for 48 h, and then infected with ASFV, ASFVΔMGF360-9L, ASFVΔMGF110-9L and ASFVΔMGF110/360-9L at a MOI of 0.1 for the indicated time points. Virus titers were measured by HAD_50_ assay. *B*, TLR2 knockdown at the indicated time points was analyzed by immunoblotting assay.

To investigate the effect of TLR2 on ASFV replication, Ma-104 cells were transfected with HA-TLR2 expression plasmid and then infected with ASFV. As shown in Figure 7*A* and Fig. S3, ectopically expressed TLR2 reduced ASFV titer in Ma-104 cells. Consistently, expression of ASFV p72 also decreased in TLR2-overexpressing Ma-104 cells in comparison with that of control cells (Fig. 7*B*). Moreover, TLR2 expression was silenced in PAMs using a small TLR2 interfering RNA (RNAi), and the results showed that TLR2 expression was markedly reduced in ASFV-infected PAM cells (Fig. 7*C*). Consequently, the ASFV titer in TLR2-knockdown cells was higher than that in control cells (Figs. 7*D* and Fig. S4) at early ASFV infection, and ASFV p72 protein expression was higher in TLR2-knockdown PAM cells than that in control cells (Fig. 7*E*). These data suggest that TLR2 inhibited ASFV replication.

## Discussion

There is a lack of information on the function of many ASFV genes. Deletions of single genes or a group of genes to obtain attenuated strains have been shown to induce protection against the virulent parental virus (14, 15, 19, 20, 22, 26, 27). In this study, *MGF110-9L* and *MGF360-9L* genes were identified as genetic determinants of virulence as their deletions of these genes highly attenuates the pathogenicity of ASFV CN/GS/2018 up to an HAD_50_ dose of 10^4^ in pigs.

Virulence may be completely abolished in the highly virulent genotype II ASFV by genetic manipulation. For instance, deletion of the *9 GL* gene (particularly potentiated by the additional deletion of the UK gene), deletion of six genes from the MGF360 and MGF530 regions, deletion of the *I177L* gene, or deletion of seven genes encoding *MGF505-1R*, *MGF505-2R*, *MGF505-3R*, *MGF360-12L*, *MGF360-13L*, *MGF360-14L*, and *E402R* caused the attenuation and complete protection of pigs against lethal ASFV challenge (3, 19, 20, 21). Our results showed that pigs infected with 10^4^ HAD_50_ of ASFV-ΔMGF110/360-9L were effectively protected when challenged at 17 dpi with the virulent parental strain with no disease-associated signs, indicating the safety of ASFV-ΔMGF110/360-9L as a potential vaccine candidate. Furthermore, it appears that replication of the challenge virus in the ASFV-ΔMGF110/360-9L–infected pigs was restricted.

The host mechanisms mediating protection against ASFV infection remain unclear. ASFV E66L inhibits host translation by the PKR/eIF2α pathway (28). DP96R of ASFV China 2018/1 negatively regulates type I IFN expression and NF-κB signaling by inhibiting both TBK1 and IKKβ, which play an important role in virus immune evasion (29). ASFV MGF360-12L inhibits type I IFN production by blocking the interaction of importinα and the NF-κB signaling pathway (30). Besides, ASFV encoding E120R protein has been confirmed to suppress type I IFN production through interacting with IRF3 (31); however, there is no evaluation of virulence and protection against parental ASFV challenge in swine by deletion of those genes. Deletion of ASFV MGF505-7R is highly attenuated in pigs at low dose by upregulating STING expression (32). I267L deficiency attenuates the virulence and pathogenesis of ASFV in pigs by impairing innate immune responses mediated by the RNA Pol-III–RIG-I axis (33); however, there is no evaluation of protection against parental ASFV challenge in swine. Analysis of different live attenuated vaccine candidates shows that there is a close association between the presence of circulating virus-specific antibodies and protection (3). However, a limited number of samples were tested; therefore, further study is required to determine whether this is the case for other vaccines.

In this study, the deletions of *MGF110-9L* and *MGF360-9L* are highly attenuated in pigs at high dose and effectively protect the pigs against lethal ASFV challenge. Generally, the protection of pigs caused by ASFV-deleted strains is multifactorial. Our previous study illustrated that *MGF110-9L* gene is transcribed at an early stage in the virus replication cycle (23) and is the inhibitor of type I IFN production (unpublished data). Besides, the upregulation of chemokines [*e.g.*, C-X-C Motif Chemokine Ligand 10 (CXCL10), C-C Motif Chemokine Ligand 2 (CCL2) and C-C Motif Chemokine Ligand 11 (CCL11)] and pro-inflammatory cytokines (*e.g.*, IL1β, IL1α, and IL13) can be observed in the ASFV-ΔMGF110-9L–infected PAMs (unpublished data), indicating the role of MGF110-9L on host innate immune response and inflammatory response. Furthermore, the difference can be found between parental ASFV and ASFV-ΔMGF110-9L infection in the regulation of MHC class II protein binding, Toll signaling pathway, apoptosis, arginine and proline metabolism and purine metabolism (unpublished data). These results implied that MGF110-9L may be involved in the regulation of multiple host functions. Our previous study also screened several host factors [Proteasome subunit alpha type (PSMA3), 26S protease regulatory subunit 4 (PSMC1), autophagy and beclin 1 regulator 1 (AMBRA1), and DEAD-box helicase 20 (DDX20)] interacted with MGF360-9L by constructing the interaction network of MGF360-9L with host proteins (34). Of these, PSMA3 and PSMC1 involved in proteasome catabolism have been verified to promote ASFV replication (34). These results indicated that MGF360-9L regulates host-cell biological processes through the proteasome pathway.

To explore the potential cause of protective phenomenon mediated by combinational deletions of *MGF110-9L* and *MGF360-9L*, functional analysis was performed, and we observed DEGs between ASFV and ASFV-ΔMGF110/360-9L–infected PAMs were mainly enriched in immune response and inflammatory response. MGF360-9L has been reported to inhibit host innate immunity by degrading STAT1 and STAT2 (24), and MGF110-9L is also an inhibitor of type I IFN production (unpublished data). Therefore, it is obvious that the enhanced innate immune response caused by ASFV-ΔMGF110/360-9L is one of a factor in the protection of pigs. We next investigate the role of inflammatory response in protection of pigs. We first observed that ASFV-ΔMGF110/360-9L induced more *Tlr2* mRNA than ASFV by RNA-Seq and qPCR assay. TLR2, one of the pattern recognition receptors expressed on the surface of immune cells, is known as a sensor of bacterial lipoproteins. Besides, TLR2 has also been reported to sense molecular patterns of viruses such as hepatitis C virus, vaccinia virus, and respiratory syncytial virus (35, 36, 37, 38, 39, 40). Engagement of the TLR2 axis leads to activation of NF-κB pathway, increased gene expression, and release of inflammation-driving mediators such as inter alia IL1β and TNFα. In this study, ASFV-ΔMGF110/360-9L enhanced Pam3CSK4-triggered TLR2 signaling compared to that of parental ASFV, indicating ASFV-ΔMGF110/360-9L induced stronger inflammatory response than parental ASFV. Finally, overexpression of TLR2 inhibited ASFV replication. Conversely, knockdown of TLR2 by RNAi in PAMs increased ASFV replication. These results demonstrated that inflammatory responses mediated by TLR2 signaling are also another factor in protection of pigs.

In summary, the Chinese ASFV CN/GS/2018 was used as a backbone to generate a virus bearing *MGF110-9L* and *MGF360-9L* gene deletions, which is highly attenuated in pigs, has a low risk of converting to a virulent strain, and could induce effective protection in pigs against lethal ASFV CN/GS/2018 challenge. ASFV-ΔMGF110/360-9L increased host *Tlr2* mRNA levels, and TLR2 inhibited ASFV replication. Our results confirmed that the deletions of *MGF110-9L* and *MGF360-9L* provide another target for the production of rationally attenuated ASFV vaccine candidates and that the attenuated virulence of ASFV-ΔMGF110/360-9L might be mediated by weakening the inhibitory effect of ASFV-ΔMGF110/360-9L on TLR2 signaling.

## Experimental procedures

### Plasmid, reagents, and RNAi

A mammalian expression plasmid for HA-tagged porcine TLR2 was constructed by standard molecular biology techniques. Rabbit anti-p65, phospho-p65, IκBα, and phospho-IκBα monoclonal antibodies (mAbs) were purchased form Cell Signaling Technology. Rabbit anti-TLR2 polyclonal antibody was obtained from Abcam. Mouse anti-Flag and β-actin mAbs were obtained from Sigma-Aldrich. Horseradish peroxidase (HRP)-conjugated anti-rabbit IgG and anti-mouse IgG were purchased from Thermo Fisher Scientific. Rabbit polyclonal antibody against ASFV p72 was prepared in our laboratory (24). TLR2 agonist PAM3CSK4, IFNβ, and IFN-γ (all from InvivoGen) were dissolved in water. The RNAi target sequence for porcine TLR2 complementary DNA (cDNA) was as follows: 5′-GCGACUUCAUUCCAGGCAATT-3′.

### Ethics statements

This study was carried out in strict accordance with the recommendations provided in the Guide for the Care and Use of Laboratory Animals of the Ministry of Science and Technology of the People’s Republic of China. All protocols were approved by the Committee on the Ethics of Animal Experiments of the Lanzhou Veterinary Research Institute of the Chinese Academy of Agricultural Sciences and the Animal Ethics Committee of Lanzhou Province, China.

### Biosafety statement and facility

All experiments involved in ASFV were conducted under the enhanced biosafety level 3 (P3) facilities in the Lanzhou Veterinary Research Institute of the Chinese Academy of Agricultural Sciences and approved by the Ministry of Agriculture and Rural Affairs and the China National Accreditation Service for Conformity Assessment.

### Cell culture, viruses, and pigs

PAMs were isolated from 30 to 40 day-old piglets as previously described (41) and maintained in RPMI 1640 medium (Thermo Fisher Scientific) supplemented with 10% fetal bovine serum (BI) at 37 °C with 5% CO_2_. ASFV CN/GS/2018 was isolated from field samples in China as described previously and stored in our laboratory (32). Ma-104 cells and iPAM cells (ATCC) were cultured in Dulbecco's Modified Eagle Medium.

Here, 50-day-old healthy Large White-Duroc crossbred pigs were obtained from local farms, with each pig being antigenically and serologically negative for ASFV, porcine respiratory and reproductive syndrome, classical swine fever, and pseudorabies virus.

### Construction of recombinant ASFV-ΔMGF110/360-9L

Recombinant ASFV-ΔMGF110/360-9L was produced by homologous recombination between ASFV-ΔMGF360-9L (24) and recombinant transfer vector. Recombinant transfer vector containing the flanking genomic regions of the MGF-110-9L gene mapping to the left (1000 bp) and right (1000 bp) of the gene and a reporter gene cassette containing the mCherry gene with the ASFV p72 late gene promoter was used (Fig. 1). Recombinant transfer vectors were generated by fusing PCR and the Gibson Assembly technique (Invitrogen Life Sciences). PAMs were transfected with the constructed transfer vector using TransITLT1 transfection reagent (Mirus Bio) and then infected with ASFV-ΔMGF360-9L at 24 h post-transfection as described previously (21). The resulting virus ASFV-ΔMGF110/360-9L was purified by successively selecting fluorescent plaques combined with limited dilution on monolayers of PAMs. Virus from the last round of purification was propagated in PAMs to obtain a virus stock. Purity of ASFV-ΔMGF110/360-9L in the virus stock was evaluated by PCR and sequencing utilizing primers MGF110-9L-F/R and MGF360-9L-F/R (Table 1).

**Table 1:** Primers used in this study

Primers	Sequence (5′–3′)
GAPDH-F	ACATGGCCTCCAAGGAGTAAGA
GAPDH-R	GATCGAGTTGGGGCTGTGACT
TNFα-F	GCCCAAGGACTCAGATCATC
TNFα-R	GGCATTGGCATACCCACTCT
IL6-F	CTGCTTCTGGTGATGGCTACTG
IL6-R	GGCATCACCTTTGGCATCTT
IRF1-F	TCCAGCCGAGATGCTAAGTG
IRF1-R	GTCCAAGTCCTGCCCGATGT
TLR2-F	GTTGCGGCTTCCAAGGATGG
TLR2-R	ATGGTCCAGAGAGTTGACCTTG
MGF110-9L-F	GAAACCGTGCAATTTATAATCCAGTCATTTTG
MGF110-9L-R	GGTGATTATTGGCCAATCATTATAAGACATTG
MGF360-9L-F	TCCATATGAACGATCTTTTGCTCGTATATTTC
MGF360-9L-R	ATGCACATGTTCTTTAAATTAATACACCCTGG

### Multistep virus growth curves

Growth curves comparing growth kinetics among the parental ASFV CN/GS/2018, recombinant ASFV-ΔMGF360-9L, ASFV-ΔMGF110-9L, and ASFV-ΔMGF110/360-9L were conducted in PAMs as described previously (17, 42). Briefly, PAMs were seeded in 48-well plates and infected at an MOI of 0.1. One hour later, the inoculum was removed and the cells were rinsed three times with PBS. PAMs were then rinsed with RPMI 1640 supplemented with 2% fetal bovine serum and incubated for 2, 24, 48, 72, and 96 h at 37 °C with 5% CO_2_. At the appropriate times postinfection, cells were collected and frozen at −80 °C, and the thawed lysates were used to determine titers by HAD_50_/0.1 ml (42) in PAMs. In this displayed experiment, three replicates were included per experimental condition.

### qPCR assay

ASFV genomic DNA was extracted from cell supernatants, tissue homogenate, or EDTA-treated whole peripheral blood using GenElute Mammalian Genomic DNA Miniprep Kits (Sigma-Aldrich). qPCR assay was carried out to amplify the conserved p72 gene segment on a QuantStudio 5 real-time PCR system (Applied Biosystems) according to the OIE-recommended procedure. A TaqMan probe (5′-[6-carboxy-fluorescein(FAM)]-ccacgggaggaataccaacccagtg-3′-[6-carboxy tetramethyl-rhodamine] from Applied Biosystems) was designed from an alignment of 54 available ASFV sequences for the 3′-end of p72. The quantity of ASFV genome was calculated by the standard curve and presented as genome copies per microliter.

### Real-time PCR (RT-PCR)

Total RNA was isolated from cells using TRIzol reagent (TaKaRa) and reverse transcribed into the cDNA utilizing PrimeScript RT reagent kit (TaKaRa). RT-PCR analysis was conducted to measure mRNA levels of tested genes. Data shown are the relative abundance of the indicated mRNAs normalized to that of GAPDH. Relative mRNA expression levels were assessed and calculated utilizing 2^ΔΔCT^ method. Gene-specific primer sequences were listed in Table 1.

### Immunoblotting assay

For endogenous immunoblotting assay, PAMs were infected with parental ASFV, recombinant ASFV-ΔMGF110-9L, ASFV-ΔMGF360-9L, or ASFV-ΔMGF110/360-9L at an MOI of 0.1 for 6, 12, and 18 h and then treated with Pam3CSK4 (100 ng/ml) for an additional 4 h. For exogenous assay, iPAM cells were transfected with Flag-MGF360-9L and HA-TLR2 plasmids for 20 h and then treated with Pam3CSK4 (100 ng/ml) for an additional 4 h. Cells were lysed in lysis buffer, and then the extracted proteins were separated by SDS-PAGE gels. Separated proteins were then transferred onto nitrocellulose membranes and probed with the following primary antibodies: TLR2 (1:250), p-IκBα (1:500), IκBα (1:500), p-p65 (1:1000), p65 (1:1000), p72 (1:2000), β-actin (1:2000), HA (1:1000), and Flag (1:1000). Subsequently, the membranes were incubated with HRP-conjugated goat anti-mouse IgG or goat anti-rabbit IgG at room temperature for 1 h. Finally, proteins were detected by chemiluminescence (ECL, New Cell & Molecular Biotech) immunoblotting substrate.

### Animal experiments

To evaluate the virulence of ASFV-ΔMGF110/360-9L in pigs, groups of 50-day-old pigs (n = 6) were i.m. inoculated with 10^4^ HAD_50_ of either parental ASFV or ASFV-ΔMGF110/360-9L. Presence of clinical signs including temperature and mortality were recorded daily for 17 days. To further evaluate the protective efficacy of ASFV-ΔMGF110/360-9L against parental virulent ASFV challenge, pigs inoculated with ASFV-ΔMGF110/360-9L were i.m. challenged 17 days later with 10^2^ HAD_50_ of parental lethal ASFV. Pigs were monitored daily for 17 days p.c. for clinical signs and mortality. Blood and tissues including the heart, liver, spleen, lung, kidney, and thymus from the dead pigs or surviving pigs, which were euthanized at the end of the observation period, were collected for ruling out the presence of ASFV using qPCR as recommended by OIE (43).

### Transcriptome sequencing (RNA-seq) and analysis

PAMs were infected with ASFV or ASFV-ΔMGF110/360-9L for 12 or 24 h, and then total RNA was extracted and used for the reference transcriptome analysis by Oebiotech. Libraries were constructed using a VAHTSTM mRNA-seq V3 Library Prep Kit for Illumina (Vazyme Inc) according to the manufacturer’s instructions. In brief, the mRNA separation/fragmentation reaction was performed, and the resulting 200 to 300 bp fragments were inserted using the hold fragmentation program at 4 °C. First strand cDNA was synthesized using random primers and reverse transcriptase, and a specific library was established when the second strand was synthesized. The library was amplified, and total DNA was screened to isolate 320 to 420 bp strands. High-throughput single-end 75 bp sequencing was performed using the NextSeq 500 Sequencing System (Illumina). Quantitative mapping was performed using the FANSe2 algorithm, and DESeq software (1.16.1) was used to screen out different genes according to the thresholds of ≥2-fold change in expression and a *p* value < 0.05. Finally, the Kyoto Encyclopedia of Genes and Genomes pathway and GO enrichment analyses were performed for the DEGs using the DAVID database.

### ELISA assay for detection of anti-ASFV antibodies

ELISA microtiter plates were coated with 800 ng per well of p30 protein in a 0.05 M carbonate buffer solution (pH 9.6) and incubated overnight at 4 °C. Antigen-coated plates were washed three times with PBS containing 0.5% v/v Tween-20 (PBST) and then blocked with 200 μl of blocking buffer (2.5% w/v nonfat dry milk in PBST) overnight at 4 °C. After three washes with PBST, 100 μl of test samples, positive serum samples, and negative serum samples diluted in blocking buffer at 1:2 were separately added to each well in duplicate. The plates were then incubated for 30 min at 37 °C followed by three washes with PBST, before adding 100 μl/well of p30 mAb-HRP (1:25,000) and incubating at 37 °C for an additional 30 min. Following a final triplicate wash with PBST, 100 μl/well of 3,3′,5,5′-tetramethylbenzidine substrate, prepared by mixing solution A (205 mM potassium citrate, pH 4.0) and solution B (41 mM 3,3′,5,5′-tetramethylbenzidine) in a 39:1 v/v ratio, was added to each well, and the plates were incubated in the dark for 15 min at 37 °C. As a final step, 2 M H_2_SO_4_ (50 μl/well) was used to stop the colorimetric reaction, and the A_450nm_ values were read using an automated ELISA plate reader.

### Statistical analysis

Results are shown as mean ± SD. An unpaired two-tailed Student’s *t* test was used to analyze the significance of the results (*p* < 0.05). All experiments were performed independently at least three times, with a representative experiment being shown.

## Data availability

All data supporting the findings of this study are available within this paper.

## Supporting information

This article contains supporting information.

## Conflict of interest

The authors declare that they have no conflict of interest with the contents of this article.
